# Supplementary material for: Antimicrobial Resistance in Qatar: Prevalence and Trends before and Amidst the COVID-19 Pandemic
Source: Antibiotics (Basel). 2024 Feb 21;13(3):203. doi: 10.3390/antibiotics13030203 (PMC10967570; doi:10.3390/antibiotics13030203)
Supplement: Supplementary file 1 [file antibiotics-13-00203-s001.zip › antibiotics-2832949-table S1.pdf]

Table S1. Spearman Rank correlation for the selected bug-drug combinations and numbers of international visitors and COVID-19 hospitalizations

Resistant Organism[1]	Number of International Visitors[2]	Number of COVID-19 Hospitalizations[2]
CRPA	-0.11 (ns)	0.4 (0.03)
ESBL-EBC	0.71 (0.0005)	-0.51 (ns)
CR-EBC	0.08 (ns)	0.05 (ns)
MRSA	0.67 (1.2×10^{-6})	-0.29 (ns)
MDRO	0.61 (0.0001)	-0.14 (ns)

[1]CRPA: Carbapenem-resistant *Pseudomonas aeruginosa*; ESBL-EBC: Extended Spectrum Beta-lactamase producing *Enterobacteriaceae*; CR-EBC: Carbapenem-resistant *Enterobacteriaceae*; MRSA: Methicillin-resistant *Staphylococcus aureus*.

[2]Spearman ρ (p-value); ns: not significant ($P > 0.05$)
