# Supplementary figures and images for: Antimicrobial Resistance in Qatar: Prevalence and Trends before and Amidst the COVID-19 Pandemic
Source: Antibiotics (Basel). 2024 Feb 21;13(3):203. doi: 10.3390/antibiotics13030203 (PMC10967570; doi:10.3390/antibiotics13030203)

Count

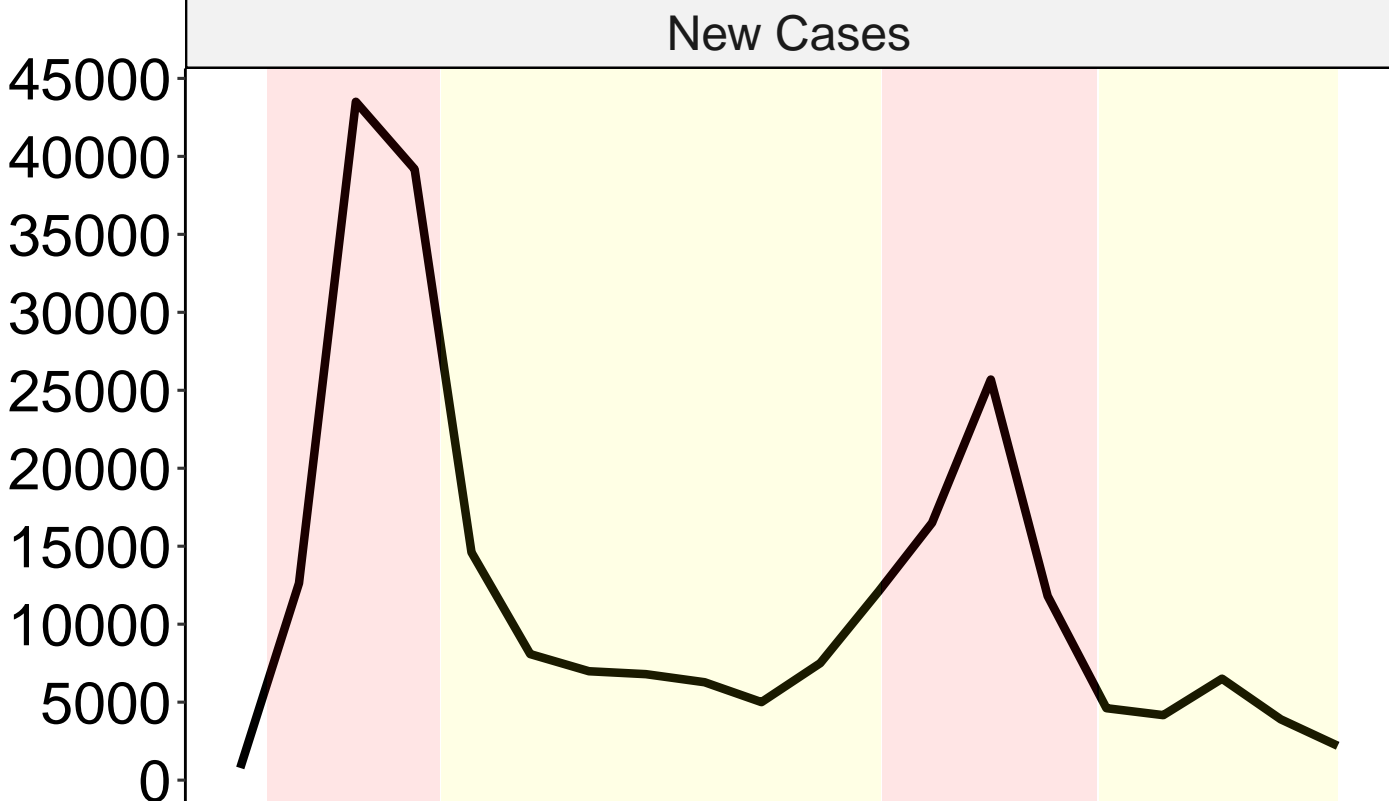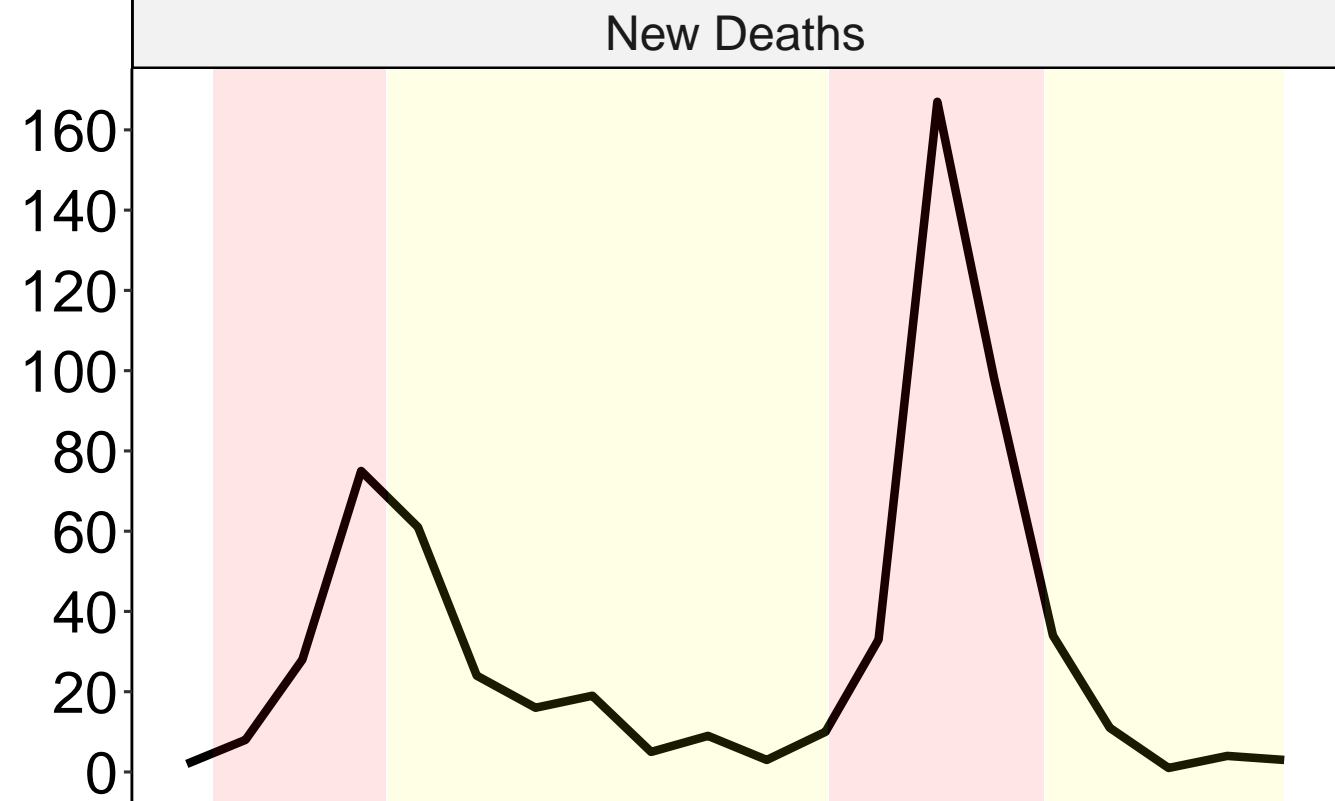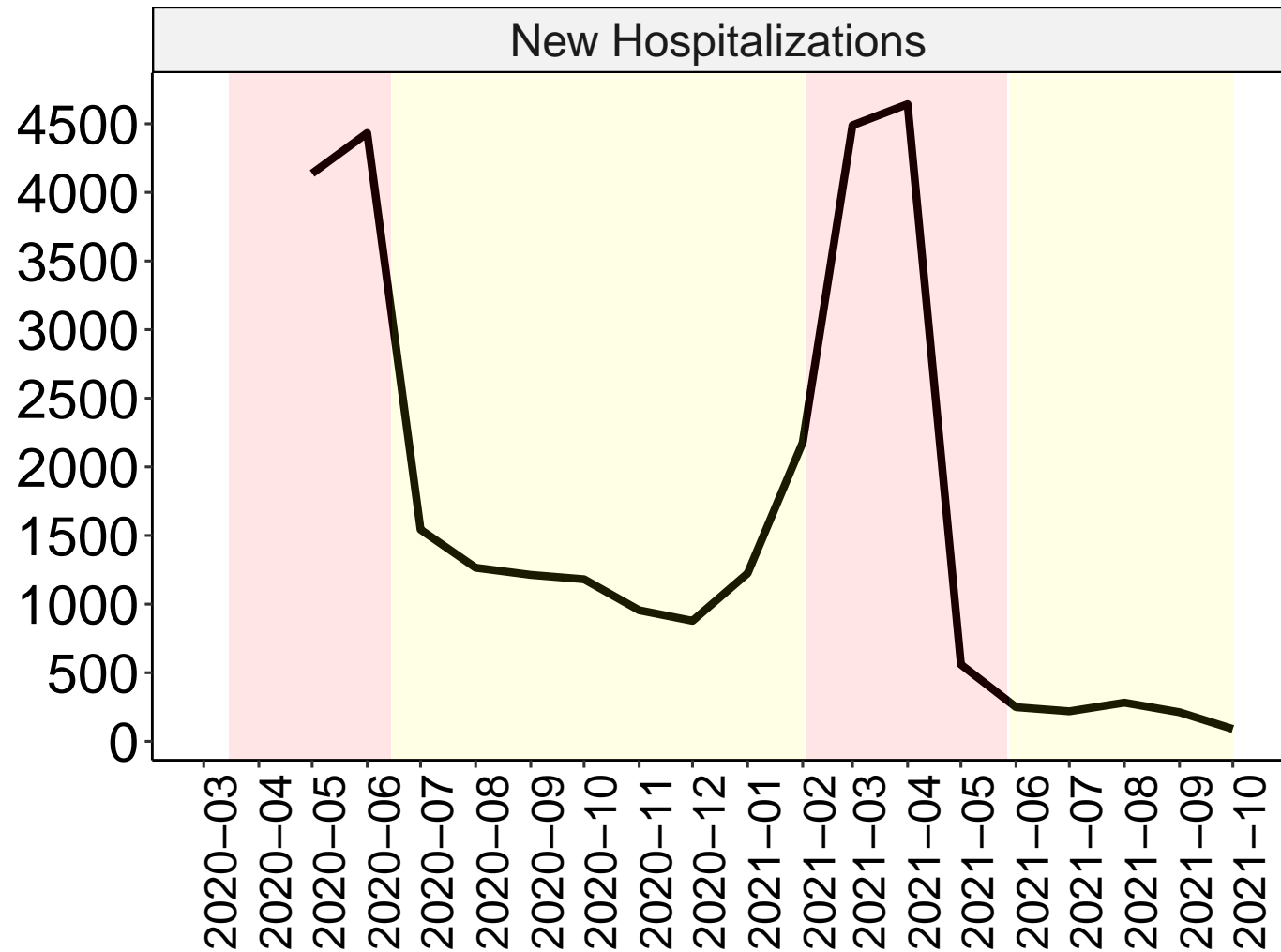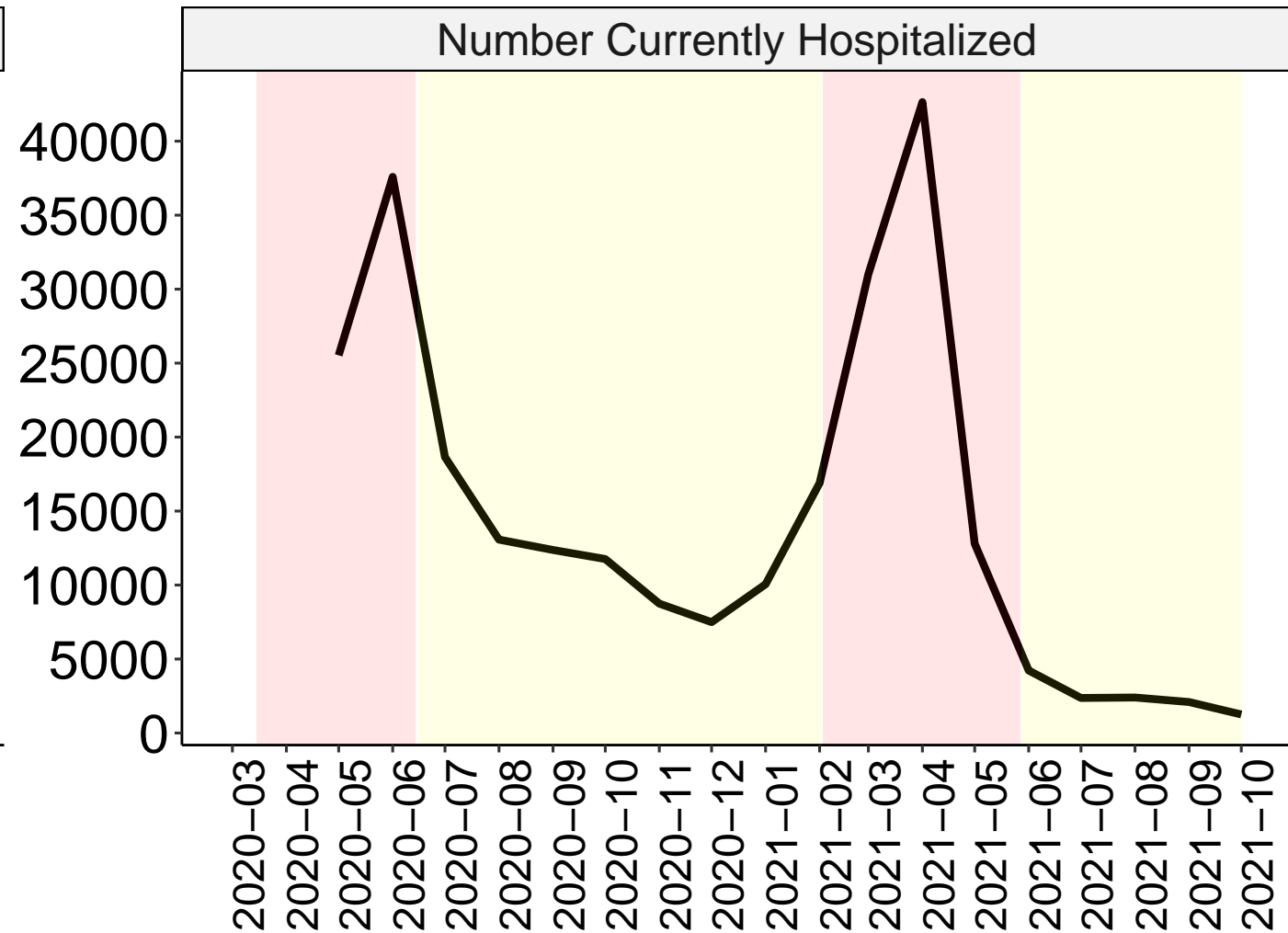

Supplement: Supplementary file 1 [file antibiotics-13-00203-s001.zip › antibiotics-2832949-figure S1.pdf]
